# Mental disorders in judicial workers: analysis of sickness absence in a cohort study

**DOI:** 10.11606/s1518-8787.2023057004737

**Published:** 2023-09-29

**Authors:** Bruna Ferreira Melo, Kionna Oliveira Bernardes Santos, Rita de Cássia Pereira Fernandes, Verônica Maria Cadena de Lima, Susan Stock

**Affiliations:** I Universidade Federal da Bahia Faculdade de Medicina da Bahia Salvador BA Brasil Universidade Federal da Bahia. Faculdade de Medicina da Bahia. Salvador, BA, Brasil; II University of Montreal Department of Social & Preventive Medicine Montréal QC Canada University of Montreal. Department of Social & Preventive Medicine. Montréal, QC, Canada

**Keywords:** Mental Disorders, Occupational Health, Mental Health

## Abstract

**OBJECTIVE:**

To analyze risk factors for sickness absence due to mental disorders among judicial workers in Bahia, Brazil.

**METHODS:**

Retrospective cohort with follow-up from 2011 to 2016 with 2,660 workers of a judicial sector in Bahia, Brazil. The main outcome measures were survival curves estimated for the independent variables using the Kaplan-Meier product limit estimator and risk factors for the first episode of sickness absence calculated based on the Cox regression model.

**RESULTS:**

The survival estimate of the population of this study for the event was 0.90 and from the Cox model the risk factors for the first episode of sickness absence due to mental disorders were: female (HR = 1.81), occupation of magistrate (HR = 1.80), and age over 30 years old (HR = 1.84). In addition, the risk for new cases of sickness absence among women reached 4.0 times the risk for men, in 2015. The estimated relative risks of sickness absence and the observed survival reduction behavior over time add information to the literature on sociodemographic and occupational factors associated with sickness absence due to mental disorders in the public sector.

**CONCLUSION:**

These results highlight the need for further research to more precisely identify vulnerable groups at risk of preventable mental health-related sickness absence in the workplace, better identify the workplace organizational factors that contribute to these disorders as well as studies on the effectiveness of workplace interventions to improve mental health among judicial and other public sectors workers.

## INTRODUCTION

Changes in the current world of work imply repercussions on working conditions with increased physical and psychosocial demands on workers and have been associated with health consequences, such as sick leaves, which are often an important public health problem^1 , 2^.

The researchers’ interest in understanding the factors associated with these occurrences in different occupational categories clarified the complexity of this event. Multiple variables can determine the context of illness and disability and stem from working conditions, the diversity of tasks required of workers, the more or less effective adaptation strategies adopted by them, as well as previous health conditions, such as comorbidities^3 , 4^.

In addition to social and economic consequences, sickness absence due to occupational disorders affects not only organizations, but also individuals, who may perceive a threat to their social participation, including job instability and even permanent exclusion from the labor market^5 , 6^. When associated with mental and behavioral disorders, sick leave is a major cause of disability for work that can lead to long term absences and make it difficult to return to work when compared with leaves for other reasons^7 , 8^.

The burden of facing the various effects triggered by this event highlights the vulnerability regarding the health condition of workers, who may be inserted in a context of social isolation^9 , 10^. Although previous studies highlight some sociodemographic, cultural, and work environment psychosocial factors that influence the occurrence or duration of mental health-related sick leave, investigating the work contexts dynamics to better understand it and intervening effectively on the preventable factors including work exposures remain a challenge for researchers, managers, and the government^11^.

Considering the lack of studies on the monitoring of mental health conditions of judicial workers, which can bring occupational and non-occupational risk factors for leaves due to mental disorders, this work intends to contribute to this discussion. Studying the complexity of the work of judicial servants and their leaves due to mental illness can help to identify possible health interventions that improve job satisfaction in these judicial organizations.

This study aimed to analyze which sociodemographic and work characteristics are associated with the risk of sickness absence due to mental disorders between 2011 and 2016 in workers from a judicial sector in Bahia, Brazil.

## METHODS

The study is a retrospective cohort during the period from January 1, 2011, to December 31, 2016, of 2,660 workers from a judicial sector in Bahia, 55.3% of whom are male and 83% have a university degree. The judicial sector covers secretariats, security posts, support centers, courts, and working offices, distributed in four municipalities in or surrounding the city of Salvador in Bahia and 28 municipalities located in the countryside of Bahia.

Work activities of the population of this study were judicial and administrative in nature, with magistrates carrying out judgment-making activities, analysts carrying out activities of reviewing court processes, and those in technical positions carrying out varied administrative activities, as well as concierges, drivers, security of property, help desk, and installation, maintenance, or support for computers, printers, and networks. The first, belongs only to judiciary departments, and the last two may vary between judiciary or administrative departments, due to an exchange of positions, common in Brazil’s public sector.

The data of sickness absence were obtained from the Information and Communication Technology Sector (ICTS) of the judicial sector. The events of sick leave due to mental and behavioral disorders were certified by doctors from the health service of the judicial sector or, when the events were certified by an external health service, they were subsequently approved by a physician from the judicial sector. Active workers were considered eligible for this study and those on sick leave due to mental and behavioral disorders at baseline of the study were excluded. Obtaining the subjects’ consent was not applicable since this study analyzed existing administrative data. The study was approved by the Research Ethics Committee of the Faculty of Medicine of Bahia under protocol No. CAAE 6309.2916.3.0000.5577 and was carried out according to the rules of Resolution 466/12 of the National Health Council, maintaining the anonymity of the subjects.

The codes identified in chapter V of the International Classification of Diseases (ICD – 10th version) were the clinical diagnoses for mental and behavioral disorders (MBD) presented at sick leave licenses, which comprised: F00-F09 (Organic and symptomatic mental disorders); F10-F19 (Mental and behavioral disorders due to psychoactive substance); F20-F29 (Schizophrenia, schizotypal, and delusional disorders); F30-F39 (Mood disorders); F40-F48 (Neurotic disorders, related to stress and somatoforms); F50-F59 (Behavioral syndromes associated with physiological disorders and physical factors); F60-F69 (Adult personality and behavior disorders); F90-F98 (Behavioral disorders and emotional disorders in childhood or adolescence); Z73.0 (Burnout).

The independent variables studied were categorized as follows: sociodemographic characteristics: sex (male and female), age (≤ 30 years old, > 30 years old), education (primary school diploma, high school diploma, university diploma); occupational characteristics: area (administrative, judicial), position (analyst, magistrate, technician), and length of service (≤ 10 years, > 10 years).

A descriptive analysis of the sickness absence profile of this cohort, with the available variables, was carried out by using proportions and cumulative incidence indicators. In addition, prevalence ratios (PR) and risk ratios (RR) were also calculated to observe the risk ratio of sickness absence due to mental and behavioral disorders due to sex, considering male as a reference category.

Survival time until the first event was measured in days until the event of interest. The records of workers who were not on sick leave during follow-up, or who left the cohort for any reason (death, transfer to another agency, dismissal, or retirement) were considered censored observations.

To estimate survival curves according to predictor factors, the “Kaplan-Meier limit-product estimator” was used^14^, and a Cox regression model was used to identify risk factors for the first episode of sickness absence due to mental health problems. For the modeling stage, only the variables gender, position, and age were considered, and the level of education was not considered due to most of the population having higher education. The variable length of service was not included due to the high correlation with age. The study population is not a random sample. Given that all workers of the judicial sector were included in the study, and the extensive literature on the appropriate use of statistical inference procedures for this situation^15^ no multivariate model variable was retained based on statistical significance. Moreover, the main criterion for selecting variables was their theoretical plausibility for potential predictors^9 , 18^.

Hazard ratios (HR) were estimated to compose the final Cox regression model and population attributable fractions for sickness absence were also calculated for each independent variable, based on absolute HR and the criteria for its utilization. To analyze the results, graphs and tables were made using Microsoft Excel 2013 program; statistical analyses were carried out with the R Studio software version 1.1.423 and the OpenEpi program.

## RESULTS

Among the 2,660 workers followed from the baseline, 262 experienced at least one event of sick leave due to mental and behavioral disorders, which shows an incidence of 10% of new sick leave cases. Of all workers, 80.80% were over 30 years old (average: 42; standard deviation: 9.74; interval: 21–69), 83.05% had completed a university degree, 58.12% worked on the judiciary department, 61.09% were in the technician position, and 77.65% had more than 10 years of service.

Among the employees on MBD sickness absence, 66.41% were female and 33.59% were male. Majority age group was over 30 years for 90.91% of men and 91.00% of women; the highest level of education completed was the university level for 72.73% of men and 86.78% of women. Regarding occupational variables, most workers were from the judicial department, 65.91% among men and 57.47% among women; in technician positions, 69.32% of men and 52.87% of women. Finally, the highest proportion of service time was over 10 years, 75% of men and 78.74% of women ( Table 1 ).

**Table 1:** Socio-demographic and occupational characteristics of workers from the judiciary of Bahia with and without mental disorder’s sickness absence, by sex, Brazil – periods 2011–2016.

Characteristic	Men				Women				Total
		
	With sickness absence by MBD		Without sickness absence by MBD		With sickness absence by MBD		Without sickness absence by MBD		−2.66
			
	n	%	n	%	n	%	n	%	
Age (years)									
≤ 30	8	9.09	176	15.13	16	9.00	234	19.00	19.20
> 30	80	90.91	987	84.87	158	91.00	1,001	81.00	80.80
Education									
Primary school	1	1.14	33	2.84	4	2.30	10	0.81	1.80
High school	23	26.13	226	19.43	19	10.92	135	10.93	15.15
University	64	72.73	904	77.73	151	86.78	1	88.26	83.05
Occupational department									
Administrative	30	34.09	478	58.90	74	42.53	534	43.08	41.88
Judiciary	58	65.91	685	41.10	100	57.47	703	56.92	58.12
Position									
Analyst	18	20.45	312	26.83	52	29.89	434	35.14	30.68
Magistrate	9	10.23	74	6.36	30	17.24	106	8.58	8.23
Technician	61	69.32	777	66.81	92	52.87	695	56.28	61.09
Service time (years)									
≤ 10	22	25.00	391	33.62	37	21.26	455	36.84	40.04
> 10	66	75.00	772	66.38	137	78.74	780	63.16	77.65

MBD: mental and behavioral disorders.

Among the employees not on MBD sickness absence, majority age group was over 30 years for 84.87% of men and 81.00% of women; the highest level of education completed was the university degree for 77.73% of men and 88.26% of women. Regarding occupational variables, most workers were from the administrative department among men (58.90%) and the judicial department among women (56.92%), with technician positions accounting for 66.81% of men and 56.28% of women. The highest proportion of service time was over 10 years, with 66.38% of men and 63.16% of women ( Table 1 ).

Workers on sick leave had 1,023 episodes of sickness absence during follow-up, with an annual average of 170.5 episodes of sick leave. There was a total of 24,806 days of sickness absence by mental and behavioral disorders at the end of six years, with an annual average of 4,134 days of sick leave per year. Regarding the incident event, the duration of the first episode of each individual had a median of 8 days and mean of 13.6 days, reaching up to 90 days in the first event.

**Figure 1:**
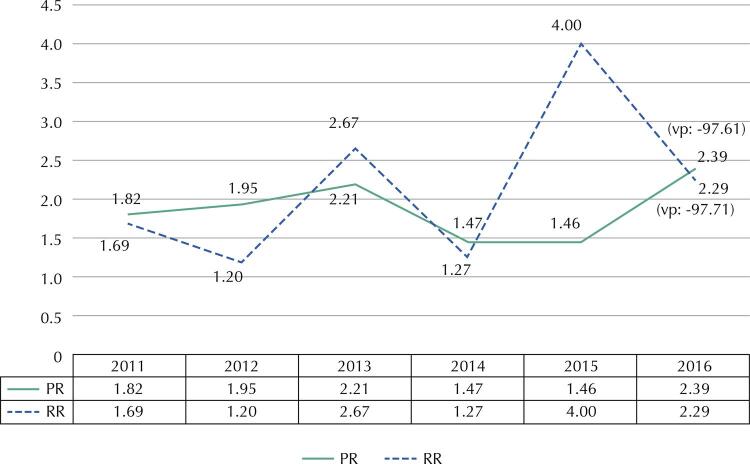
Risk ratios (RR) and prevalence ratios (PR) of mental disorders sickness absence of workers by sex in the judicial sector of Bahia.

Higher risk for cases of sickness absence due to mental disorders was observed among females compared with males, indicating a possible sex-related exposure component. In 2013 and 2015, female workers had 2.7 times and 4.0 times the risk of sickness absence when compared with men, respectively. In the more recent years of follow up, an upward trend in risk of events was also observed among women when compared with men ( Figure 1 ).

VP: variação percentual proporcional.

In the Cox model, the time until the first episode of sickness absence was considered as the outcome. An increased risk for sick leave was shown in relation to the independent variables studied of gender, age, and position ( Table 2 ). Differences between subgroups within the analyzed categories were not found in the modeling stage.

**Table 2:** Cox multiple model for sickness absence due to mental disorders among judicial workers from the judiciary of Bahia, Brazil – periods 2011–2016.

Characteristic	Crude hazard ratio	Adjusted hazard ratio	Population attributable fractions
		
	HR	HR	HR
Sex			
Female	1.76	1.81	0.29
Male	1	1	-
Position			
Magistrate	1.95	1.80	0.08
Analyst / technician	1	1	-
Age (years)			
> 30	1.93	1.84	0.44
≤ 30	1	1	-

HR: hazard ratio.

Note: adjusted by all model variables.

In this multiple analysis, females had an 81% higher probability of sickness absence than males (adjusted HR = 1.81). Among the magistrate workers, the adjusted risk was 80% higher compared with analysts and technical personnel (HR = 1.80), and among workers over 30 years old, the adjusted sickness absence rate was 84% higher when compared with younger workers (HR = 1.84) ( Table 2 ). The greater population attributable fractions were due to higher age (44%) and female sex (29%). In this population, 8% of work absence for MBD was attributable to the occupation of magistrate.

The estimated overall survival of this population for mental and behavioral disorders sickness absence was 0.902 (approximately 90%), according to the Kaplan-Meier estimate (survival rate). The analysis of Kaplan-Meier curves generally showed slightly different survival patterns between stratifications, since over time, survival decreased for all exposure categories among the predictor variables analyzed.

**Figure 2:**
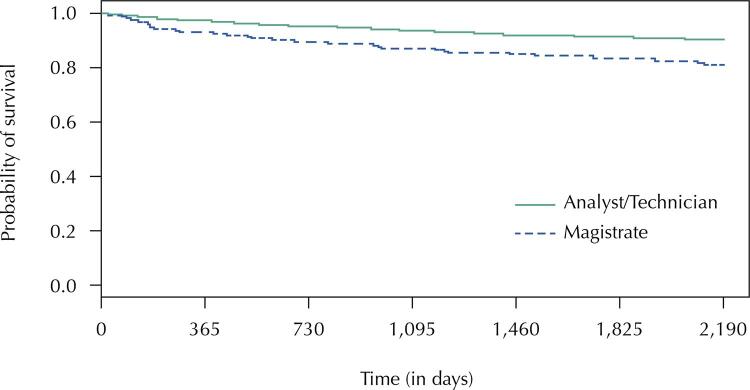
Survival curves estimated by Kaplan-Meier considering mental disorders sickness absence for the occupation variable.

Figure 2 shows the survival graph by job category. A higher proportion of magistrates reported sickness absence due to mental and behavioral disorders at some point during the six-year study period compared with analysts and technical personnel (17.8% *versus* 9.1%).

## DISCUSSION

This study found that, in a population of judicial civil servants in the state of Bahia, the risk of sickness absence due to mental or behavioral disorders is higher among females, workers older than 30 years, and magistrates compared with analysts and technical personnel.

The cut-off point adopted for the age group in this study was 30 years old for better description of results, despite the risk of sickness absence by mental disorders increasing with age. Moreover, the highest population attributable fraction (44%) was due to this variable. Advancing age and the presence of comorbidities have been suggested as factors that predict episodes of long-term sickness absence for mental health problems^19 , 20^. The combination of older age and the occurrence of mental disorders may also reflect a pattern of early retirement or may be related to greater difficulties in returning to work^13^.

As a new finding, our study showed that magistrates had a higher risk of mental and behavioral disorder sickness absence and shorter survival for the event over a 6-year follow up period. Mental health problems in judges have been described and attributed to various sources of stress that may characterize their work context^21^. The daily life of magistrates can involve high workload and high psychological work demands, associated with the volume of trials to be judged; the strain of presiding over hearings with high stakes disputed interests, that is, dealing with two conflicting parties; requiring making decisions on verdicts that will impact the lives of others; and even with the fear of possible reprisals^20^. Despite their high social status, the tasks performed by magistrates reflect work environments characterized by very high burdens of responsibility and intense emotional demands, similar to that of other overcommitted occupations such as doctors and police^22^.

High incidence rates of sickness absence for mental or behavioral disorders appear to be associated with emotionally demanding work activities that confront workers with daily situations of conflict, strain, or fear that represent a kind of psychological overload^23^. In addition, the isolation required to maintain impartiality in decisions may be associated with working in a solitary fashion and having low levels of social support, which may in turn lead to mental exhaustion and burnout for these professionals^22^.

This study showed that magistrates had 80% more episodes of sick leave due to mental or behavioral disorders compared with analysts and technicians. To our knowledge, no other empirical studies explored specific predictors of sickness absence in judicial workers or in the judicial sector. Furthermore, little is known about the psychosocial work demands of this occupational category and their work exposures. Some previous studies have studied the relation between magistrates and offenders with mental disorders^24 , 25^.

The lack of research addressing occupational stressors of magistrates reinforces the importance of directing attention to this category, both to identify conditions that may lead to work exhaustion or mental health disorders in magistrates and to identify potential workplace interventions to prevent mental health-related sickness absence and evaluate their effectiveness.

In addition to the psychosocial work context related to the work of magistrates, discussing the higher risk of mental health sickness absence found generally among female workers is also useful. Of all sickness absence in the study population, 29% could be attributed to the female sex. This may be related to very high work demands as well as the double burden of family and domestic responsibilities many female workers face^26^. Besides, throughout the cohort a positive risk of sick leave was observed in women in comparison to men. Most sick leaves in women may be explained by this gender’s characteristic of searching for health services^27^. However, longitudinal follow-up of the cohort for a longer period of time is needed.

Future research on the risks associated with sickness absence in women should more explicitly explore these hypotheses and include exposures related to work environments, social life, family life, the health system, and working conditions such as remuneration.

In terms of ergonomic conditions, women showed a major incidence of the event in magistrate workers, with an occupation with higher demand, but also work control, stability, and possibility of social ascension. In addition, men showed a greater number of sick leaves in technician position, which may reveal the vulnerability of mechanical occupations, typically occupied by men, compared with occupations with greater cognitive expenditure to the occurrence of this event.

This study measured the time until the first episode of sickness absence for mental and behavioral disorders to calculate the risk rate. Other studies in the literature consider the recurrence of sickness absence due to mental and behavioral disorders as the outcome, since it is an event with chronicity characteristics and high likelihood of recurrence. These studies, therefore, calculate indicators that consider each recurrent sickness absence as an incidence episode^28^. Others, however, consider only new cases in the numerator, *i.e.* , the first event for each worker with a mental health-related sickness absence, the same procedure adopted in this study.

Although the analysis of recurrences allows the observation of the event’s dynamics in the population, the model to identify associations with the first episode of sickness absence may allow discovery of latent characteristics of the individual and the organization associated with the triggering event^9^.

The absence of work organization and other work exposure variables in our model, which unfortunately are almost never measured in the administrative data, limited the analysis of occupational predictors of sickness absence due to mental and behavioral disorders in this population. Also, due to an insufficient number of cases in the database, the categories needed to be dichotomized for age and education variables. More refined categories might have given different results. Future epidemiologic research should include organizational and psychosocial exposures associated with judicial work, given what is already known about high work demand and low work control in this setting which may influence sickness absence^3^.

The combination of different psychosocial factors, such as rotation of different tasks and functions, difficulties with required skills, lack of internal support, or conflicts in interpersonal relationships, create a potential exposure context for psychological overload and sickness absence. In addition, the onset of mental health disorders in an occupational environment may occur for other reasons, such as disability due to musculoskeletal diseases or reduced participation in work by sickness absence due to other diseases^3 , 12^.

Finally, the results of this study demonstrated a profile of workers on sick leave similar to that of other longitudinal studies that investigated sickness absence for mental and behavioral disorders, with a predominance of women, over the age of 30^7 , 22 , 28^.

## CONCLUSION

This study analyzed the risk factors for the incidence of sick leave due to mental and behavioral illnesses in a retrospective cohort of judicial servants. Despite considering limitations due to the reduced number of variables available in the database, the study shows a higher risk of sickness absence among women and magistrates. This study highlights the need to further study the organizational context and social environment of magistrates’ work as well as the personal factors, domestic context, larger social environment, and access to support and treatment that may contribute to these sickness absences with multidisciplinary qualitative and epidemiologic studies. Once the contributing factors are more clearly identified, appropriate preventive interventions in the workplace can be developed, implemented, and evaluated for effectiveness. This information can be applied to foster new research including surveillance to monitor workers’ mental health and associated working conditions to identify vulnerable workplace groups and eventually implement evidence-based changes in public service management practices and other preventive actions to reduce workplace problems among judicial workers.
